# Enrichment of intracellular sulphur cycle –associated bacteria in intertidal benthic foraminifera revealed by 16S and *aprA* gene analysis

**DOI:** 10.1038/s41598-019-48166-5

**Published:** 2019-08-12

**Authors:** I. S. Salonen, P. -M. Chronopoulou, C. Bird, G. -J. Reichart, K. A. Koho

**Affiliations:** 1 University of Helsinki, Faculty of Biological and Environmental Sciences, Ecosystems and Environment Research Program, P.O. Box 65 (Viikinkaari 1), FI-00014 University of Helsinki, Helsinki, Finland; 2 University of Stirling, Biological and Environmental Sciences, FK9 ALA, Stirling, United Kingdom; 3 Department of Ocean Systems, NIOZ-Royal Netherlands Institute for Sea Research and Utrecht University, Den Burg, The Netherlands; 4 Department of Earth Sciences – Geochemistry, Faculty of Geosciences, Utrecht University, P.O. Box 80.021, 3508 TA Utrecht, The Netherlands

**Keywords:** Biogeochemistry, Ocean sciences, Microbial ecology

## Abstract

Benthic foraminifera are known to play an important role in marine carbon and nitrogen cycles. Here, we report an enrichment of sulphur cycle -associated bacteria inside intertidal benthic foraminifera (*Ammonia* sp. (T6), *Haynesina* sp. (S16) and *Elphidium* sp. (S5)), using a metabarcoding approach targeting the 16S rRNA and *aprA* -genes. The most abundant intracellular bacterial groups included the genus *Sulfurovum* and the order Desulfobacterales. The bacterial 16S OTUs are likely to originate from the sediment bacterial communities, as the taxa found inside the foraminifera were also present in the sediment. The fact that 16S rRNA and *aprA* –gene derived intracellular bacterial OTUs were species-specific and significantly different from the ambient sediment community implies that bacterivory is an unlikely scenario, as benthic foraminifera are known to digest bacteria only randomly. Furthermore, these foraminiferal species are known to prefer other food sources than bacteria. The detection of sulphur-cycle related bacterial genes in this study suggests a putative role for these bacteria in the metabolism of the foraminiferal host. Future investigation into environmental conditions under which transcription of S-cycle genes are activated would enable assessment of their role and the potential foraminiferal/endobiont contribution to the sulphur-cycle.

## Introduction

Benthic foraminifera are unicellular eukaryotes widespread across marine environments. Due to their high abundance and predominance in the benthic ecosystem, they play an important role in the sedimentary carbon cycle by participating in phytodetritus processing and organic matter uptake^1,2^. Living at and also deeper within the sediment implies that these foraminifera sometimes live under oxygen depleted conditions and potentially rely on alternative biogeochemical pathways^3^. Benthic foraminifera are known to actively take part in the nitrogen cycle, as several species have the ability to take up and store nitrate intracellularly^4–7^ and to perform complete denitrification^4,8^. A recent genome analysis of two *Globobulimina* species suggests the existence of a novel denitrification pathway encoded by foraminifera’s own genome^8^. In certain areas, benthic foraminifera may even be responsible for the majority of the benthic denitrification process, which highlights their global importance in the nitrogen cycle^9,10^. In addition to carbon and nitrogen cycling, recent evidence of foraminiferal sulphur uptake in labelling experiments^11^ suggests that foraminifera may also play a role in the sedimentary sulphur cycle.

Benthic foraminifera are known to harbour a range of potential bacterial endobionts, including putative denitrifying bacteria and sulphur-oxidizing bacteria^12–14^. Recently, methanotrophs were also found to be associated with benthic foraminifera^15^. As such, the function of the putative endobiont community may be diverse, ranging from metabolic strategies to the ability to inhabit otherwise hostile environments, such as dysoxic, sulphidic sediments^13,16^. Endosymbiotic relationships are also common in other marine eukaryotes, offering them potential evolutionary benefits, as they help the host to adapt to unstable conditions and survive in unfavourable environments^17^. For example, ciliates are known to harbour a variety of endobionts linked to carbon, nitrogen and sulphur cycles, which are crucial for the survival of the host species^16,18^. In ciliates, the endosymbiotic relationships are known to have developed independently and species-specifically, and they persisted on long geological time scales^19^. The origin of the benthic foraminiferal endobionts is currently not well understood. It has been suggested that they may be transferred from generation to generation^14^. Alternatively, they may be drawn from the ambient sediment, similar to planktonic foraminifera, which are suggested to have evolved their endosymbioses via interactions with water column bacteria^20^. However, so far very little is known of the interactions between foraminifera and the surrounding sediment bacterial community.

Sedimentary bacterial communities may play a role in foraminiferal diet. However, bacterial uptake by benthic foraminifera has been shown to be unselective, implying that bacterivory occurs mainly in association with potential deposit-feeding behaviour^21^. Feeding strategies, as well as organic matter turnover rates, appear to be species-specific^21–23^. For example, *in situ* experiments with ^15^N labelled bacteria have shown that benthic foraminifer *Ammonia tepida* prefers algae in its diet over bacteria^24^, suggesting that bacteria are not its primary food source. In addition to bacterivory, foraminifera are known to have a variety of other feeding strategies, such as herbivory, carnivory and even direct dissolved organic carbon uptake^21,23,25,26^. Mixotrophy is also an important trophic strategy for some shallow-water benthic foraminifera with the ability to harbour photosymbionts or kleptoplasts^27–29^. The photosymbiont associations can be diverse and flexible, and it has even been suggested that some species are able to shuffle their photosymbionts to increase adaptability^30^. Kleptoplast-driven photosynthesis and associated inorganic carbon fixation is shown to be an important carbon sequestration mechanism for *Haynesina germanica*^31^. Additionally, kleptoplasts may serve as an energy reservoir under unfavourable conditions^32^.

Despite the significant contribution of these ubiquitous and abundant organisms to both C and N cycling e.g.^1,2,4,8–10^ very little is known of the potential interactions between the sediment bacteria and benthic foraminifera. Previously, endobiont studies have been mainly based on transmission electron microscope (TEM) observations^33^ and lacked direct comparisons to the sediment microbial community. Recently, 16S rDNA metabarcoding has provided insights into intracellular bacterial communities of planktonic foraminifera, allowing the identification of putative species-specific endobionts^20,34^. Here, we use a metabarcoding approach to target the 16S rRNA gene and focus on 3 benthic species, *Ammonia* sp. (T6), *Elphidium* sp. (S5), and *Haynesina* sp. (S16)^23^ collected from intertidal localities in the Dutch Wadden Sea. We compare the 16S rDNA metabarcoding –derived intracellular bacterial operational taxonomic units (OTUs) to those of the ambient sediment to determine which bacterial groups are enriched within foraminifera, and link the findings to sediment porewater chemistry and sediment bacteria distribution. We examine potential species-specific intracellular bacterial 16S OTUs in foraminifera, as well as, the effect of sediment depth and sampling location. Furthermore, the sulphur cycle-related *aprA* (dissimilatory APS reductase) functional gene is quantified and sequenced, to explore the potential for intracellular bacteria-driven sulphur oxidation/sulphate reduction in benthic foraminifera, and to study the phylogenetic relationships of the associated bacteria.

## Results

### Pore water geochemistry

**Figure 1:**
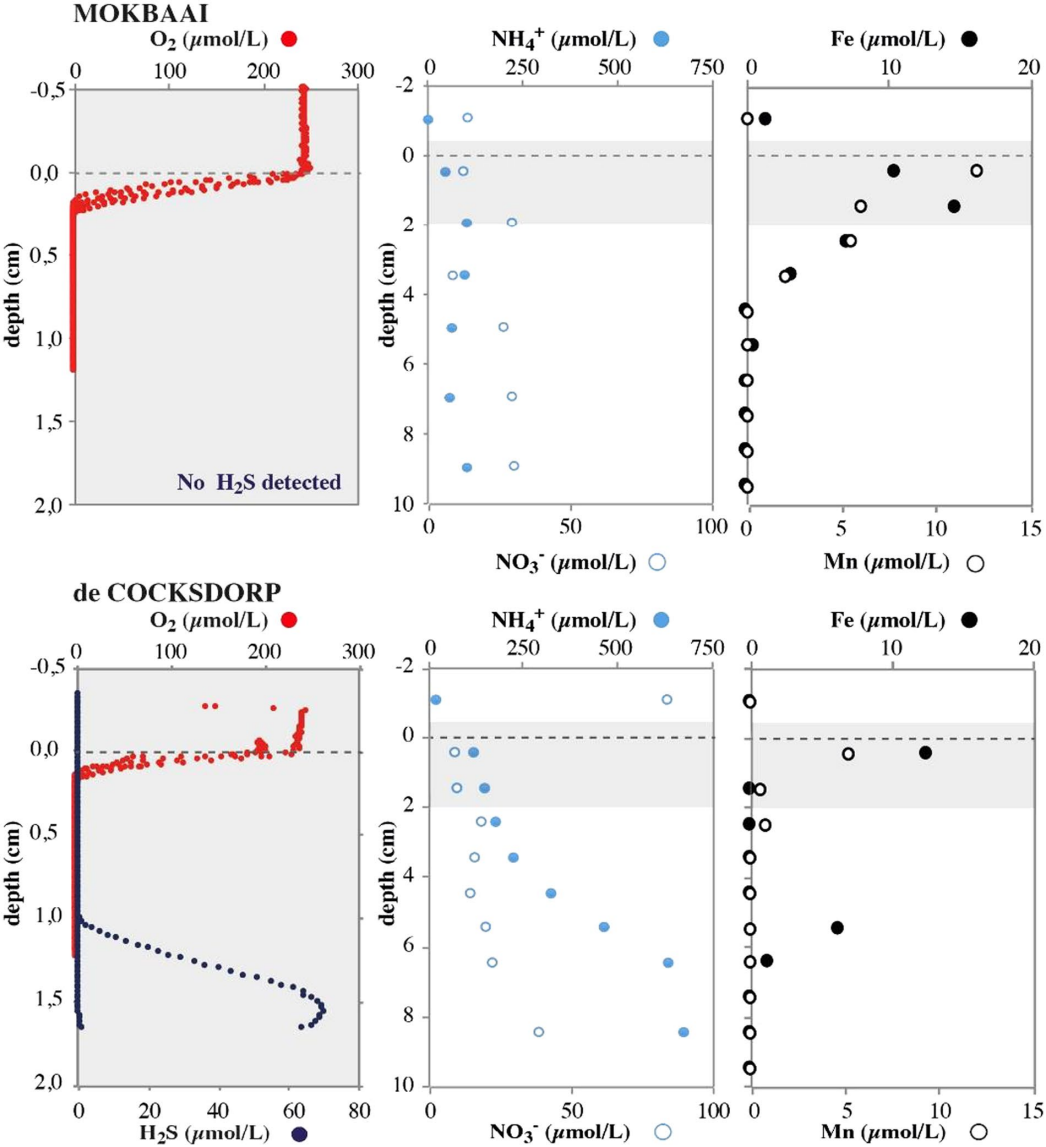
Porewater profiles of O_2_ and H_2_S, NH_4_^+^ and NO_3_^−^, Fe and Mn at both Mokbaai and de Cocksdorp.

The oxygen penetration depth at both de Cocksdorp and Mokbaai sites was approximately 0.2 mm (Fig. 1). Below the oxygen penetration, a clear manganese (Mn) and iron (Fe) reduction zone was detected, indicated by increases in the availability of dissolved Mn and Fe. In Mokbaai, the Fe and Mn concentrations in the surface sediments were close to 20 µmol/l, declining to zero at approximately 4 cm sediment depth. At de Cocksdorp, the decline is faster, and the concentration of Fe and Mn dropped to 0 µmol/l before 2 cm sediment depth. A small Fe peak at approximately 5 cm sediment depth may be related to bioturbation. No clear denitrification zone was detected in pore water nitrate (NO_3_^−^), with NO_3_^−^ being present, both in Mokbaai and de Cocksdorp, down to 10 cm sediment depth (Fig. 1). Pore-water ammonium (NH_4_^+^) was clearly higher in de Cocksdorp than in Mokbaai, where an increase to over 600 µmol/l was seen at 10 cm sediment depth, suggesting relatively higher remineralisation of organic matter (OM) in de Cocksdorp compared to the Mokbaai sediments. The enhanced OM remineralisation in de Cocksdorp was also evident from the presence of H_2_S in surface sediments, whereas no H_2_S was detected in Mokbaai at the depths sampled.

### Intracellular foraminiferal- and sediment-based bacterial 16S OTUs

Three species were retrieved from Mokbaai, including 13 *Ammonia* sp. (T6), 9 *Elphidium* sp. (S5) and 1 *Haynesina* sp. (S16); and 5 from de Cocksdorp, of which all were *Elphidium* sp. (S5) specimens (Supplementary Table S1). The 16S Illumina MiSeq sequencing produced a total of 4 019 303 sequences in the sediment dataset and 7 097 136 sequences in the foraminiferal dataset. The number of sequences after trimming and quality filtering was 423 845 and 937 601, and resulted in 16 255 and 16 356 OTUs in the sediment and foraminiferal datasets, respectively. After filtering OTUs with low number of reads (total sum of reads per OTU across all samples <5 in foraminiferal dataset, <10 in sediment, Supplementary Fig. S1), the number of OTUs was further reduced to 2521 (sediment) and 1896 (foraminifera). Trimming down the OTU number reduced the total number of sequence reads by 8.9% (sediment) and 2.4% (foraminifera). Levelling rarefaction curves indicate that the sequencing depth was satisfactory in sediment samples (Supplementary Fig. S2). Foraminiferal specimens varied more in the amount of reads and OTUs obtained (Supplementary Fig. S2).

**Figure 2:**
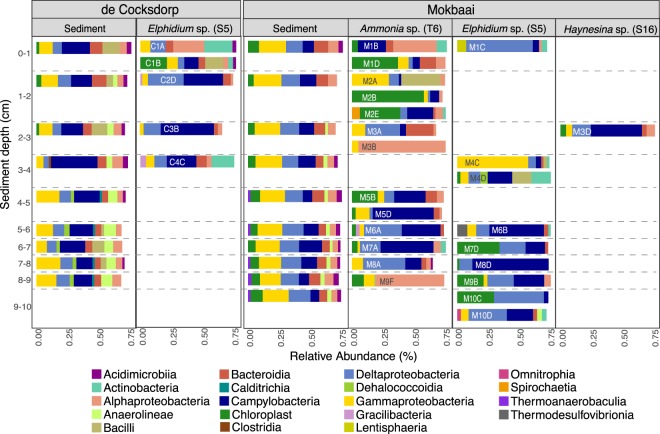
Relative abundance (%) of bacterial classes (≥2%) in sediment and in foraminifera samples.

**Figure 3:**
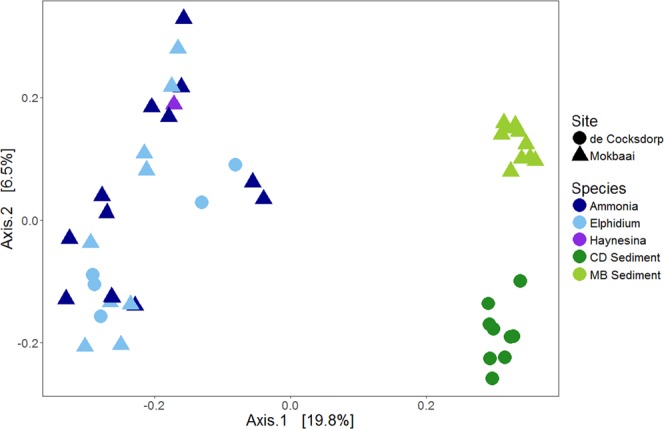
Principal coordinate analysis (PCoA) including both sediment and foraminifera samples.

**Figure 4:**
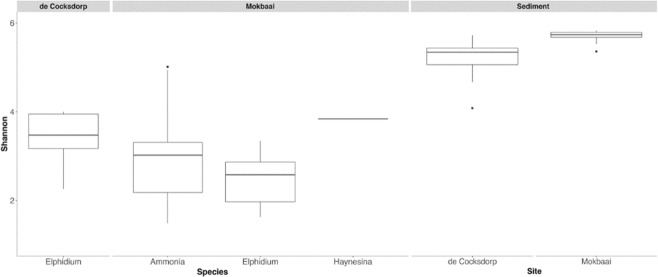
Shannon diversity index (H’) in foraminifera and sediment samples in box-whisker plot. Lines indicate median value, boxes standard error and error bars the standard deviation.

The number of bacterial classes was similar in sediment samples (88) and inside foraminifera (89). The intracellular bacterial 16S OTUs of foraminifera consisted of similar bacterial classes to those found in the sediment but at contrasting relative abundancies (Fig. 2). The difference between the foraminiferal intracellular 16S OTUs and the sediment was clearly identified by principal coordinate analysis (PCoA), using Bray-Curtis distance where the sediment and foraminiferal specimens were separated on the x-axis, explaining 19.8% of variance (Fig. 3). Alpha diversity (Shannon index) was higher in sediment (median 5.7 Mokbaai, 5.3 de Cocksdorp) than inside the foraminifera (median 2.6 to 3.8 at Mokbaai, 3.4 at de Cocksdorp) (Fig. 4).

**Figure 5:**
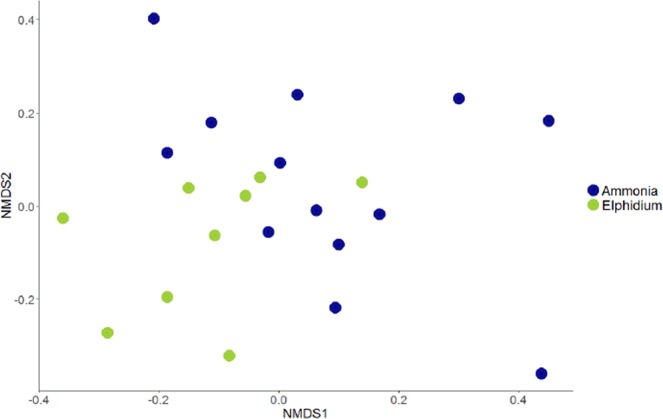
Non-metric multidimensional scaling (nMDS) plot of *Ammonia* sp. (T6) and *Elphidium* sp. (S5) from Mokbaai.

The sediment bacterial 16S OTUs at de Cocksdorp were dominated by class Campylobacteria (up to 44.3% relative abundance, Fig. 2). Campylobacteria was also the most abundant class inside the foraminifera (all *Elphidium* sp. (S5)) at this site, but with a higher relative abundance, making up to 51.5% of the 16S OTUs (Fig. 2). The most common genus of this class in both sediment and foraminifera (up to 99.6% of all Campylobacteria reads) was the sulphur-oxidizing bacterium (SOB) *Sulfurovum* (Supplementary Table S3). In addition, at de Cocksdorp, classes such as Deltaproteobacteria, Actinobacteria and Chloroplasts were more relatively abundant in foraminifera (up to 36.8%, 29% and 27.4%, respectively) than in sediments (up to 12.7%, 1.3% and 4.7%, respectively). At Mokbaai, Campylobacteria dominated the intracellular bacterial 16S OTUs of foraminifera (up to 63.1% in *Ammonia* sp. (T6) 79.1% in *Elphidium* sp. (S5) and 50.1% in *Haynesina* sp. (S16) (Fig. 2), whereas in the sediments bacterial classes Gammaproteobacteria (up to 25.7%) and Deltaproteobacteria (up to 20.2%) were the most abundant (Fig. 2). Differences were observed in the intracellular bacterial 16S OTUs among the 3 species. For example, *Ammonia* sp. (T6) contained a higher relative abundance of Alphaproteobacteria (up to 90.3%) than *Elphidium* sp. (S5) (up to 6.8%) or *Haynesina* sp. (S16) (8%), whereas *Elphidium* sp. (S5) had more Deltaproteobacteria (up to 69.7%) than *Ammonia sp*. (T6) (up to 43.8%) or *Haynesina* sp. (S16) (18.2%) (Fig. 2). In addition, chloroplast OTUs were detected in the intracellular bacterial OTUs of some foraminiferal specimens (up to 74.5%). SILVA classification was not able to distinguish the source of chloroplasts, however, additional BLAST search implied that majority of the most common chloroplast OTUs in the sediment and inside foraminifera were originated from diatoms (Supplementary Tables S4 and S5). The closest BLAST match of the most abundant intracellular chloroplast OTU of foraminifera (in average 59.8 ± 25.3% in all foraminifera) was a chloroplast isolated from a benthic foraminifera *Virgulinella fragilis*^14^ (Supplementary Table S4). Other common chloroplast OTUs were mainly from diatom sources, and some of them, such as *Triceratium dubium*, *Extubocellus spinifer* and *Plagiogramma staurophorum* were also common chloroplast OTUs in the sediment (Supplementary Tables S4 and S5). According to non-metric multidimensional scaling analysis, Mokbaai (excluding *Haynesina* sp. (S16), as only one specimen of this species was available), *Ammonia* sp. (T6) and *Elphidium* sp. (S5) specimens were separated on the x-axis (NMDS1) (Fig. 5). Indeed, species was found to be a significant factor (p-value = 0.004, PERMANOVA), whereas sediment depth was not (p > 0.1, PERMANOVA).

**Figure 6:**
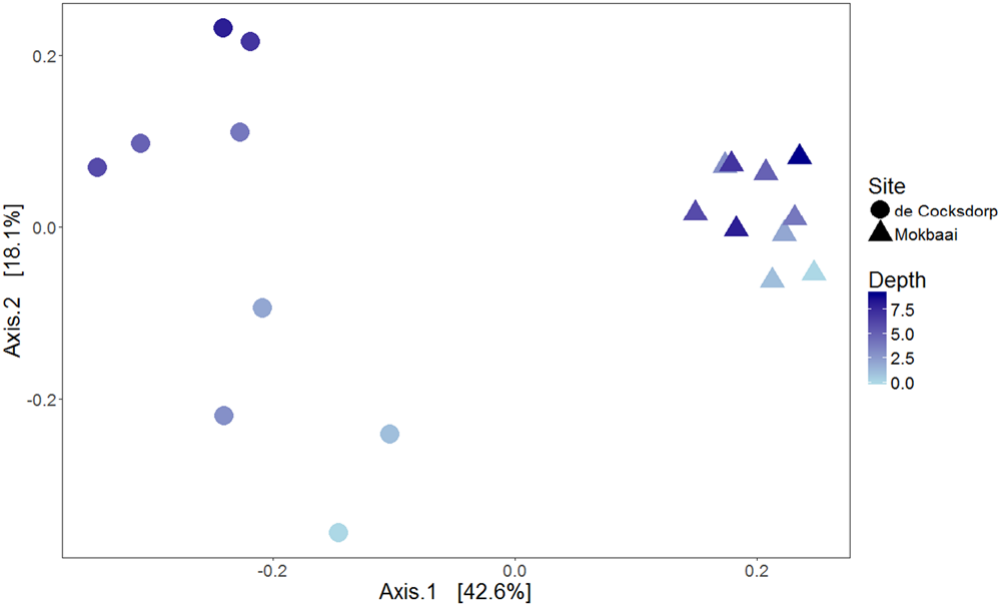
Principal coordinate analysis (PCoA) of sediment samples showing in-sediment depth in color.

Sediment bacterial communities at the two sites were different, as shown in the principal coordinate analysis (PCoA), where the sites are separated on the x-axis (42.6% of the variance, Fig. 6). This separation is likely due to contrasting relative abundancies of the dominant taxa, for example at Mokbaai bacterial classes Gammaproteobacteria (up to 25.7%) and Deltaproteobacteria (up to 20.2%) were dominating, whereas at de Cocksdorp, class Campylobacteria was more abundant (up to 44.3% in de Cocksdorp, 21.2% in Mokbaai) (Fig. 2). In addition, chloroplasts (up to 11.3%) were more abundant at Mokbaai. The sediment community also changed with depth, as indicated by the y-axis of the PCoA plot (18.1% of the variance, Fig. 6). At de Cocksdorp, Bacilli (16.4% to 0.01%) and Chloroplasts (2.7% to 0.8%) decreased with depth whereas anaerobic classes, such as Anaerolinae (1.9% to 10.6%) and Dehalococcoidia (0.02% to 4%), increased. At Mokbaai, the anaerobic classes Anaerolinae (1.2% to 3.9%) and Thermoanaerobaculia (1.2% to 2.3%) also increased with depth (Fig. 2). In the sediment, the driving factors for the variance in the bacterial communities were site (p-value 0.001, PERMANOVA) followed by sediment depth (p-value 0.004, PERMANOVA). In contrast, when looking at *Elphidium* sp. (S5), which was found at both study sites in 0–4 cm depth, neither site nor depth was found to be significant (p > 0.1, PERMANOVA). Thus, the overdriving factor determining the sediment bacterial community was site followed by depth, whereas the composition of intracellular bacterial 16S OTUs of foraminifera was species-dependent.

### Intracellular aprA OTUs of foraminifera

**Figure 7:**
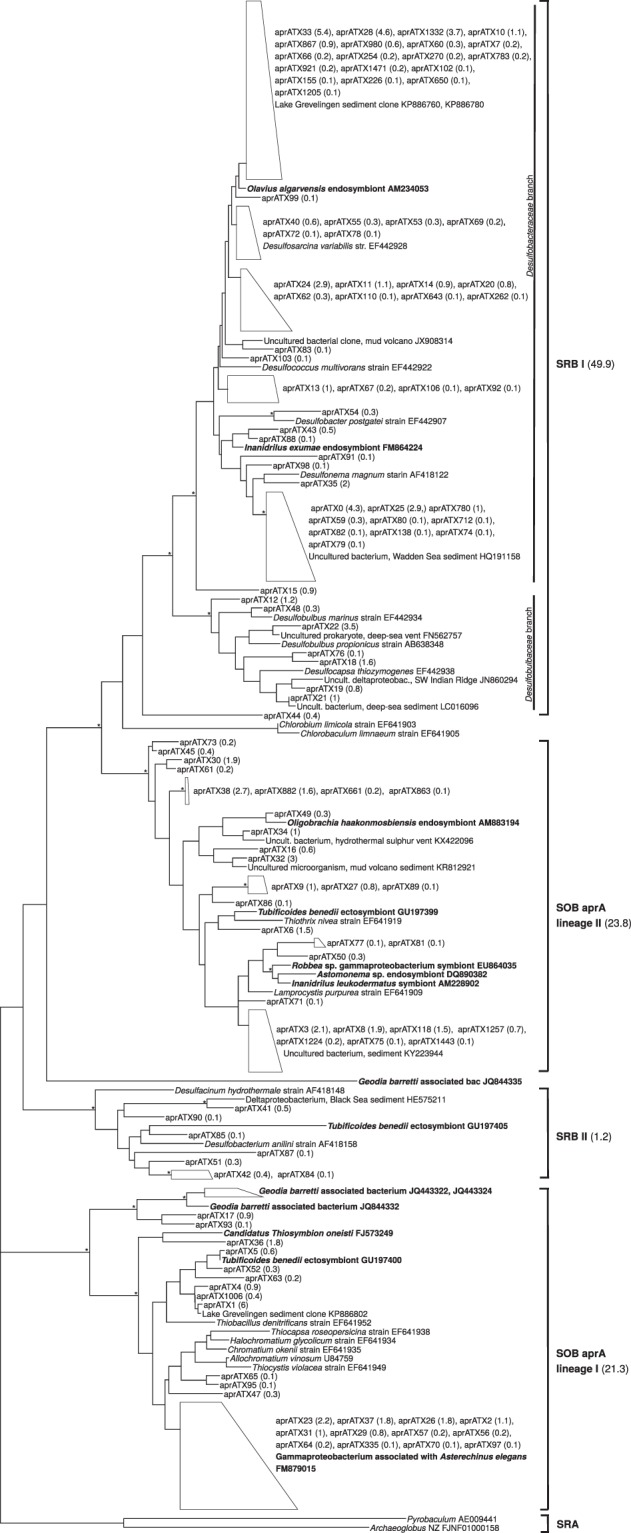
Phylogeny based on ML analysis of the partial *aprA* gene (about 370 bp). Collapsed branches are indicated by a polygon. Bootstrap values over 70% are shown with an asterisk (*). OTUs of this study are marked with aprATX prefix, with the relative abundance of that OTU in average across all samples in parenthesis. Known symbionts are indicated in bold. SRB: sulphate-reducing bacteria; SOB: sulphur-oxidizing bacteria; SRA: sulphate-reducing archaea.

In foraminifera, the abundant class Campylobacteria was dominated by the SOB genera *Sulfurovum* and *Arcobacter* (up to 95.7% and 40.3% of reads inside the class, respectively). All foraminifera species also harboured a high relative abundance of Deltaproteobacteria (up to 43.8% in *Ammonia* sp. (T6), 69.7% in *Elphidium* sp. (S5) and 18.2% in *Haynesina* sp. (S16)), in which common genera included sulphate-reducing bacteria (SRB), such as, *Desulfobacula*, *Desulfosarcina* and *Desulforhopalus* (Supplementary Table S3). In total, 687 sulphur-cycle related intracellular *aprA* OTUs (referred to as aprATX in Fig. 7) of foraminifera were analysed.

Phylogenetic analysis (Fig. 7) confirms the relation of intracellular foraminiferal *aprA* OTUs to both SOB and SRB. In fact, the analysed intracellular foraminiferal *aprA* OTUs are almost evenly distributed between SOB (45.1% of all sequences) and SRB (51.1% of all sequences) (Fig. 7). Four well defined clusters can be seen on the tree: 2 SRB clusters (SRB I, consisting of 49.9% of all sequences; SRB II, consisting of 1.2% of all sequences) and 2 SOB clusters (SOB *aprA* lineage I with 21.3% of all sequences; SOB *aprA* lineage II with 23.8% of all sequences) (Fig. 7). The SOB clusters have 91% (SOB *aprA* lineage II) and 96% (SOB *aprA* lineage I) ML bootstrap support. The most abundant OTU (aprATX1, relative abundance 6% across all specimens) is found in SOB *aprA* lineage I and is related (98.9% BLAST similarity) to a sediment isolate of the saline Lake Grevelingen in the Netherlands^35^. More sequences from this lake are part of the largest branch on our tree, comprising 19 OTUs (18.5% of all reads), which is clustering with known *Desulfobacteraceae*. A close relative of this group of sequences (89–90% BLAST similarity) is an endosymbiont of the oligochaete *Olavius algarvensis*^36^. Few *aprA* OTUs group together with symbiotic bacteria (Fig. 7). For example, the lowest branch of the SOB *aprA* lineage I, consisting of 9.6% of *aprA* OTUs groups together with a Gammaproteobacterium associated with the echinoid *Asterichinus elegans* gut microflora^37^. In addition, single *aprA* OTUs, such as aprATX6 (1.54% of reads), clusters with a *Tubificoides benedii* ectosymbiont in SOB *aprA* lineage II^38^, and aprATX17 and aprATX93 (1% of reads) cluster with the marine sponge *Geodia barretti*-associated bacteria^39^ in the SOB *aprA* lineage I (Fig. 7).

**Figure 8:**
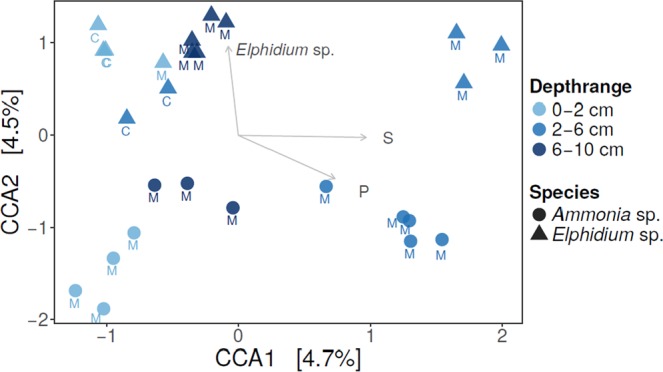
Canonical correspondence analysis (CCA) of intracellular *aprA* bacterial OTUs. Arrows showing explanatory variables. C = de Cocksdorp, M = Mokbaai.

As with the bacterial 16S OTUs, each foraminiferal species appeared to have distinct bacterial *aprA* OTUs. According to canonical correspondence analysis (CCA), based on *Ammonia* sp. (T6) and *Elphidium* sp. (S5) from both sites (*Haynesina* sp. (S16) excluded due to lack of adequate replicates), species was a significant factor influencing the intracellular *aprA* OTUs (p-value 0.026, ANOVA) whereas site was not (Fig. 8). Quantification of the S cycle-related genes (*aprA* and *dsrB*) showed that the gene copy numbers were high across specimens and sites (Supplementary Table S6). In sediments, the *aprA* gene copy numbers were 2.1 × 10^7^ (SD 1.4 × 10^7^) and 2.9 × 10^7^ (SD 1.8 × 10^7^) per gram dry sediment at de Cocksdorp and Mokbaai, respectively. Per single foraminiferal cell, the *aprA* gene copy numbers were on average 2.3 × 10^2^ (SD 2.4 × 10^2^) in *Ammonia* sp. (T6), 3.7 × 10^2^ (SD 3.5 × 10^2^) in *Elphidium* sp. (S5), and 8.4 × 10^1^ in *Haynesina* sp. (S16) (Supplementary Table S6). In contrast, the quantification of N cycle-related genes was not consistent across samples, and their amplification was unsuccessful with the exception of the *nirS* gene (Supplementary Table S6). The *nirS* gene was sequenced on the Illumina MiSeq platform, alongside blank samples. The resulting communities were similar to the blank samples, which were dominated (over 99% of total *nirS* reads) by a single OTU 89% similar to Gammaproteobacterium B9-12 (AJ248393) from Pacific NW sediments. Thus, we reason that no real *nirS* community was captured.

## Discussion

The 16S rDNA metabarcoding revealed a wide diversity of both SRB and SOB enriched among the intracellular bacterial 16S OTUs of foraminifera compared to the surrounding sediment bacterial community. Although, some intraspecific variation was observed (Fig. 2), statistically intracellular bacterial 16S OTUs as well as *aprA* OTUs of foraminifera were species-specific. Overall, alpha diversity was lower in foraminifera compared to sediments, which could potentially imply a selective uptake, although differences in the amount of sample material (0.25 g sediment *vs*. a single foraminiferal cell) may also be the driver of the lower diversity obtained. The genetic potential for sulphur oxidation and sulphate reduction was further identified by targeting and quantifying the *aprA* gene, which was found to be abundant across different foraminiferal species. In contrast, intracellular N-cycle associated bacteria were not successfully targeted in foraminifera, implying that they play a trivial role in our specimens compared to S-cycle associated bacteria. We were able to amplify a rather long DNA fragment (approximately 550 bp) of intracellular bacterial DNA from foraminifera, implying that the bacterial DNA was not degraded by digestion, which would typically limit the length of the fragments obtained^40^. As such, the intact nature of the extracted DNA implies that the bacteria inside the foraminifera may be alive and putatively endobiotic. Intact and dividing bacteria have also been previously observed inside intertidal benthic foraminifera *Ammonia* sp. (T6), suggesting that putative bacterial endobionts could exist at least in this species^41^. To verify the activity and function of the putative endobionts, RNA and/or FISH analysis are recommended.

The presence of bacterial OTUs inside the foraminifera solely due to bacterivory cannot be completely excluded but seems very unlikely. Although similar bacterial taxa were present in the foraminifera and the surrounding sediment, these occurred in contrasting relative abundancies. Foraminifera digest bacteria randomly^21^ while deposit feeding, which would likely result in intracellular bacterial composition that would more closely mirror that of the ambient sediment. Random deposit feeding would also not be expected to result in species-specific bacterial 16S OTUs observed here. In addition, previous work has shown that bacterivory plays only a minor role in fulfilling the carbon requirements of benthic foraminifera^21,24,42^. Instead, the elphidiid specimens (*Elphidium* sp. S5, *Haynesina* sp. S16), are kleptoplastic^29^ and thus are likely to have a dietary preference for diatoms^23^, whereas *Ammonia* sp. T6 has been suggested to exhibit also carnivorous behaviour^23,43^. The relatively low yield of chloroplasts in elphidiids in this study, compared to the study of Chronopoulou *et al*. (2019) where universal 18S primers were used to target eukaryotes on the same specimens, may be related to the limited ability of 16S primers to target these organelles. Thus, to better target intracellular algal signal we recommend the use of universal eukaryotic primers. As an alternative to being solely a food source, the sediment bacterial community could also provide endobionts to benthic foraminifera in a similar way that the endobionts of pelagic foraminifera have been linked to the surrounding water column^20^.

Previous studies have suggested that SOB could potentially have an endobiotic relationship with foraminifera^14^. In addition, sulphur (^34^S) incorporation under dysoxia was observed for *Ammonia* sp. (T6), implying an ability to potentially synthesize sulpholipids through a sulphate activation pathway^11^. In other marine eukaryotes, endo- and ectobionts associated with the sulphur cycle are widespread in marine environments and occur in several phyla^17^. The host can benefit from them in various ways. For example, SOB symbionts can fix carbon autotrophically while deriving energy from sulphur-oxidation, and provide the host with organic carbon sources^17^. Sulphur-oxidizing symbioses have been discovered in marine sponges^39^, nematodes^44^, ciliates^18,45,46^ and oligochaete worms^36,47^. In turn, SRB symbionts can produce sulphide by oxidizing either organic compounds e.g. acetate or inorganic compounds e.g. hydrogen. Some eukaryotes, such as the oligochaete worm *Olavius algarvensis*, can even harbour both SOB and SRB, forming an endosymbiotic sulphur cycle, potentially helping the host cope with sediments with variable sulphide concentrations^36,47^. In our data, several *aprA* OTUs in the SRB I branch cluster together with the *O. algarvensis* Delta 1 symbiont. Overall, the sulphur cycle –related OTUs were almost equally distributed between SRB and SOB branches, in which they grouped with known symbiotic bacteria of other marine eukaryotes, suggesting that some of the *aprA* OTUs could be putative endosymbionts for the foraminifera. These closely related symbionts included, for example, ectosymbionts of the oligochaete worm *Tubificoides benedii* isolated from the Wadden Sea coastal sediments^38^. In the SRB I branch, the foraminiferal intracellular bacterial OTUs contained a large abundance of sulphate-reducing Deltaproteobacteria, belonging to bacterial families *Desulfobulbaceae* and *Desulfobacteraceae*. In ciliates, these same bacterial taxa have been identified as endobionts growing autotrophically and potentially providing the host with amino acids^18^. Similarly, kleptoplast-bearing foraminifera are able to receive photosynthates, such as amino acids, directly from their symbionts^48^. In addition, utilisation of dissolved amino acids has been observed in benthic foraminifera^25^, although the exact mechanism for this is poorly understood. We suggest that sulphur-cycle related endobionts could potentially benefit foraminifera by providing carbon or other vital compounds, such amino acids, to the host.

Dynamic environments with changing redox conditions, such as intertidal mudflats, have been estimated to be a potential hotspot for sulphur-cycle related symbiotic associations^38^. They are in general characterized by a very shallow oxygen penetration depth^49^ and variable redox stress due to non-steady state porewater geochemistry associated with tidal activity and bioturbation. In this study, at de Cocksdorp, the sediment became oxygen depleted after 0.2 mm and free H_2_S was detected below 1 cm sediment depth (Fig. 1). Despite the challenging conditions, foraminifera can thrive in these environments due to their ability to survive long periods of anoxia^5,50,51^ and tolerate sulphidic conditions^52^. In the Wadden Sea, all three species are commonly encountered and contribute significantly towards benthic biomass^1,22,42^. Under anoxia, the metabolic rate of foraminifera decreases^53^ and their cytoplasm gets thinner^41^. However, despite reduced metabolism, foraminifera must still sustain their vital functions and have been shown to continue to grow and calcify^54^. In other eukaryotes, such as ciliates, endobionts are hypothesized to be crucial for survival in anoxic/dysoxic environments^18,45^. In a similar way, endobionts could help benthic foraminifera to adapt to changing environmental conditions. The relatively diverse composition of intracellular bacterial 16S OTUs may also provide the foraminifera an advantage in responding to environmental stress, as they could potentially utilize the most appropriate endobiont community, in a similar fashion to photosymbiont-bearing foraminifera that have been suggested to potentially shuffle their internal symbiont pool in response to changes in environmental conditions^30,55^. In addition to sulphur-cycle bacteria, the intracellular bacterial 16S OTUs observed in this study included chloroplasts, of which the most abundant one was similar to kleptoplastic endobionts previously isolated from *Virgulinella fragilis*^14^. It has been suggested that harbouring chloroplasts along with SOB symbionts may have the advantage of reducing the harmful effects of H_2_S^12,14^, which could benefit kleptoplastic species such as *Elphidium* sp. and *Haynesina* sp. The genetic and metabolic diversity of putative sulphur cycle-associated endobionts might help foraminifera to colonize unstable, dynamic environments, where oxygen is limited but sulphate and sulphide is abundant.

## Conclusion

To date, sulphur-cycle related putative endobionts in benthic foraminifera have been largely overlooked and understudied, however, as our data shows, the genetic potential for both sulphur oxidation and sulphate reduction is abundant in the studied foraminiferal species from two different locations. Furthermore, these SOB and SRB are phylogenetically closely related to known symbiotic bacteria of other marine eukaryotes. We therefore hypothesize, that these putative endobionts, which foraminifera may derive from the ambient sediment, could be linked to foraminiferal carbon / nutrient acquisition, allowing the foraminifera to inhabit the periodically anoxic and sulphidic intertidal sediments. Future studies targeting the activity of the putative endobionts are needed to confirm their functions and roles in foraminiferal ecology.

## Methods

### Study sites and sampling

The samples were collected in November 2015 from two sites situated on intertidal mudflats on the coast of Texel island, the Netherlands. The sediment at Mokbaai (53°00′17.2″N 4°45′22.6″E) appeared generally sandier and during sampling large polychaete worms and burrows were observed to >10 cm sediment depth. At de Cocksdorp (53°09′23.2″N 4°52′53.0″E), situated on the north side of Texel, polychaete worms and large burrow structures were absent.

From both sites, an intact sediment core (inner diameter 10 cm) was retrieved during low tide. Both cores were transported immediately to the Netherlands Institute of Sea Research (NIOZ), which is located within 5 km distance from Mokbaai and 20 km distance from de Cocksdorp, for further processing. In a temperature-controlled laboratory (set at 12 °C), dissolved oxygen and hydrogen sulphide (H_2_S) were measured, using Unisense microelectrodes. Porewater oxygen profiles were measured with a Unisense microsensor (OX-100), two-point calibrated in 100% air-saturated filtered sea water collected from the study site, and in an anoxic solution, containing sodium ascorbate and NaOH (both at 0.1M). Oxygen measurements were carried out at depth intervals of 100 µm. The pore water H_2_S profiles were measured with a Unisense (H2S-100) microsensor, four-point calibrated in an anaerobic solution containing Na_2_S at concentrations of 0, 12.5, 25, 50 µmol/L (according to manufacturer’s instructions), and measurements were carried out at depth intervals of 200 µm.

After profiling, the core was subsampled with 3 truncated syringes, of which 2 were used for pore water analysis and 1 for both environmental DNA (eDNA) sequencing and picking of the foraminifera. The two syringes for porewater measurements were immediately placed in a nitrogen-flushed glove bag and the sediment was sliced at 1 cm intervals down to 10 cm depth. Slices were transferred into 50 ml centrifuge tubes fitted with 0.45 μm maxi-spin centrifuge filters and subsequently centrifuged at 3000 rpm for 20 minutes outside the glove bag. Afterwards, the tubes were transferred back into the glove bag, where the supernatant was filtered (0.25 μm) and divided into subsamples. The nutrient analyses of NO_3_^−^ and NH_4_^+^ were carried out at the Royal NIOZ according to standard protocols^56,57^, respectively. The elemental samples (namely iron and manganese) were acidified with 1M HNO_3_ and measured with ICP-OES at the University of Helsinki, Department of Food and Environmental Sciences (precision and accuracy <5% RSD as determined by in-house and reference standards). All values are reported as μmol L^−1^.

The third syringe was also sliced with 1 cm intervals down to 10 cm sediment depth. Each slice was first subsampled for sediment eDNA with a sterile plastic spatula (1–1.5 g of sediment frozen in liquid nitrogen and stored at −20 °C). The remaining sediment was sieved with filtered seawater through a 125 µm mesh and living foraminifera were collected based on motility^41^. Foraminifera were identified to genus level based on morphology, which was verified with 18S V9 amplicon sequencing^23^. All specimens were washed minimum 3 times using sterile artificial sea water (ASW), to remove any remaining sediment from their shell. Afterwards, foraminifera were placed in RNA*later* (ThermoFischer) solution, in order to dissolve their carbonate shells whilst keeping the nucleic acids intact, and stored at +4 °C until further molecular analyses.

### DNA extractions and sequencing

The carbonate-free foraminiferal cells were carefully washed again a minimum of 3 times with sterile ASW to remove all remains of their shell and residual RNA*later*^20,34^. The clean, intact cells were crushed into DOC buffer for DNA extraction^58^. Sediment DNA was extracted from 0.25 g of sediment with the PowerSoil® DNA Isolation Kit (MoBio, Carlsbad, USA) according to the manufacturer’s instructions. To analyse the 16S gene, DNA was amplified with the Polymerase Chain Reaction (PCR) method, using a mixture of the universal bacterial primers pA_Illum_FP_1-3 and pD′_Illum_RP_1-3 targeting the V1-V3 regions of the 16S rRNA gene^59^ (Supplementary Table S2). The PCR conditions were as follows: 98 °C for 10 seconds (s), followed by 32 cycles (foraminifera) or 25 cycles (sediment) of 98 °C 5 s for denaturation, 72 °C 15 s for annealing and 72 °C 30 s for elongation and 72 °C for 1 minute (min) for final elongation. Each PCR product was visualized with gel electrophoresis (1% agarose) to check if a single band of the correct size was observed and the PCR was successful. In order to control potential contamination, to which 16S rDNA metabarcoding of a single cell is susceptible, blank samples were used alongside foraminiferal specimens (DOC buffer and ASW), and sediment samples (extraction kit buffers), as well as non-template blanks for PCR and post-PCR.

The PCR purifications, second PCR round and Illumina MiSeq sequencing were performed in the Laboratory of DNA sequencing and Genomics in the Institute of Biotechnology at the University of Helsinki^59^. Unique custom barcodes for later sample de-multiplexing were selected using BARCOSEL^60^. After sequencing, raw reads were sorted into samples based on barcodes. Then, MiSeq overhangs, barcode and primer sequences were removed^59^. 16S rDNA sequences were assembled to paired-end reads and quality-filtered in Mothur version 1.36.1, according to the MiSeq Standard Operating Procedure^61^. Maximum length was set to 550 base pairs (bp). Quality filtered reads were aligned against the SILVA database (release 132) and chimeric sequences were removed in Mothur with the UCHIME tool^62^. OTUs were created using 97% similarity threshold. Taxonomy was assigned in Mothur against the SILVA database using representative sequences corresponding to the distance centroids of each OTU. The blanks were analysed alongside samples and the OTUs that were abundant in the blanks (consisting of 99.6% reads in the blanks) were subsequently removed from the final 16S rDNA dataset. Finally, in order to avoid diversity overestimation while preserving our sequencing effort, OTUs summing up to <5 (foraminifera specimens) and <10 (sediment samples) sequence reads were removed. These thresholds were set based on plotting the cumulative sum of OTUs that would be filtered against the total counts (Supplementary Fig. S1).

To quantify common nitrogen cycle genes (*amoA*, *nirS*, *nirK*, *norB;* Supplementary Table S2) and sulphur-cycle genes (*aprA, dsrB;* Supplementary Table S2) we performed quantitative PCR (qPCR) (Supplementary Table S6). The same specimens were used for the qPCR analysis as for the 16S rDNA metabarcoding. The reaction for each specimen was performed in triplicate in a final volume of 10 μl, which contained 5 μl of SensiFAST SYBR No-ROX mix (2x) (Bioline), 200 nmol/L of each primer and 1 μl of 10 times diluted DNA. The conditions for all reactions were as follows: 95 °C for 3 min; 40 cycles of 95 °C for 5 s and 60 °C for 30 s; 95 °C for 5 s; 65 °C for 5 s, and a final step of 95 °C for 30 s. Absolute quantification of the targeted genes was performed with a series of 10-fold standard dilutions, using the CFX Manager (version 4.0) software (Bio-Rad). Standards were derived from environmental purified PCR products. Following quantification, the functional gene adenosine-5′-phosphosulfate reductase (*aprA*) was amplified and sequenced to target bacteria involved in the sulphur cycle (Supplementary Table S2). The PCR conditions were as follows: a denaturation step at 98 °C for 30 s, 20–28 cycles of 98 °C for 10 s, 72 °C for 15 s for annealing, 72 °C for 15 s, and a final elongation at 72 °C for 1 min. Sequencing was done on the Illumina MiSeq platform as described earlier with the 16S gene. Processing of the *aprA* sequences was done in the QIIME pipeline (version 1.9.1) and its associated modules^63^. OTUs were specified at 90% similarity level using the USEARCH algorithm^64^ and taxonomy of the most abundant representative sequences was assigned by a BLAST search^65^ against the National Center for Biotechnology Information (NCBI) database.

To construct the phylogenetic tree, representative sequences of the most abundant *aprA* OTUs (i.e. OTUs of ≥0.05% relative abundance) were aligned with their closest relatives (85–100% similarity) and *aprA* sequences of known sulphur-cycle bacteria, and known endo- or ectosymbiotic sulphur cycle –associated bacteria from other marine eukaryotes, such as sponges, oligochaetes and nematodes. Alignment was done using the muscle algorithm^66^ (version 3.8.31) and edited in MEGA7^67^. Maximum likelihood (ML) phylogenetic tree was constructed using MEGA7, after performing a “best model” analysis to select the best substitution model (Tamura 3-parameter model with discrete Gamma distribution rates among sites) according to BIC (Bayesian Information Criterion)^67,68^. The tree was edited in Dendroscope^69^ (version 3.5.9) and Adobe Illustrator CC (version 23.0.2).

### Statistical analysis

Statistical analysis was done in R (version 3.4.2). Alpha diversity (Shannon) and rarefaction analysis was calculated using package vegan^70^ (version 2.4-5). Non-metric multidimensional scaling analysis, principal coordinate analysis and canonical correspondence analysis were done using packages phyloseq^71^ (version 1.22.3) and ggplot2^72^ (version 3.0.0). Significance of variables was determined using PERMANOVA function in vegan^70^.

## Supplementary information

Supplementary information

## Data Availability

The representative DNA sequences of OTUs of the 16S sequence data are available in NCBI GenBank under accession numbers MK646075 - MK647970 (foraminifera) and KCXS00000000, PRJNA528017 (sediment). The representative DNA sequences of *aprA* OTUs (aprATX) are available in NCBI GenBank under accession numbers MK569530 - MK569654.
