# Supplementary material for: Enrichment of intracellular sulphur cycle –associated bacteria in intertidal benthic foraminifera revealed by 16S and aprA gene analysis
Source: Sci Rep. 2019 Aug 12;9:11692. doi: 10.1038/s41598-019-48166-5 (PMC6690927; doi:10.1038/s41598-019-48166-5)
Supplement: Supplementary file 1 — Supplementary information [file 41598_2019_48166_MOESM1_ESM.docx]

Supplementary information

Enrichment of intracellular sulphur cycle –associated bacteria in intertidal benthic foraminifera revealed by 16S and *aprA* gene analysis

Salonen, I. S.^1*^, Chronopoulou, P-M.^1^, Bird, C.^3^, Reichart, G.-J.^4, 5^, Koho, K. A.^1^

1 University of Helsinki, Faculty of Biological and Environmental Sciences, Ecosystems and Environment Research Program, P.O. Box 65 (Viikinkaari 1), FI-00014 University of Helsinki, Finland

3 University of Stirling, Biological and Environmental Sciences, FK9 ALA, Stirling, United Kingdom

4 Department of Ocean Systems, NIOZ-Royal Netherlands Institute for Sea Research and Utrecht University, Den Burg, the Netherlands

5 Department of Earth Sciences – Geochemistry, Faculty of Geosciences, Utrecht University, P.O. Box 80.021, 3508 TA Utrecht, The Netherlands

* Corresponding author, email: iines.salonen@helsinki.fi


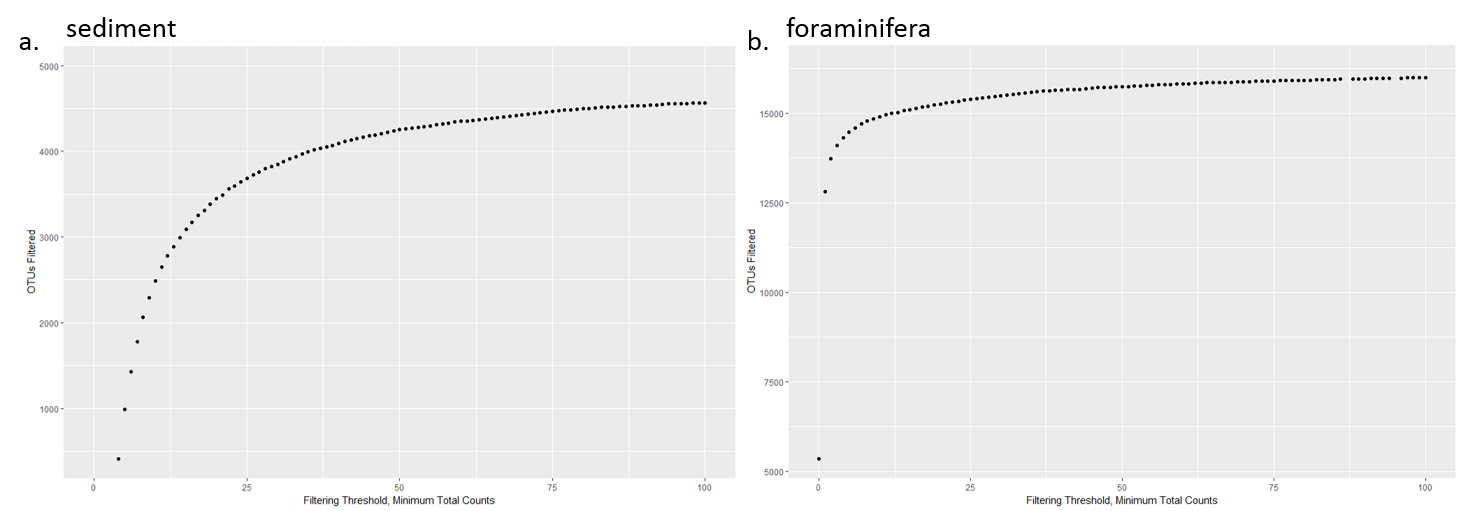


Fig. S1 Scatter plot of OTUs versus total counts, used to determine the cutoff for sediment (a) and foraminifera (b) datasets.


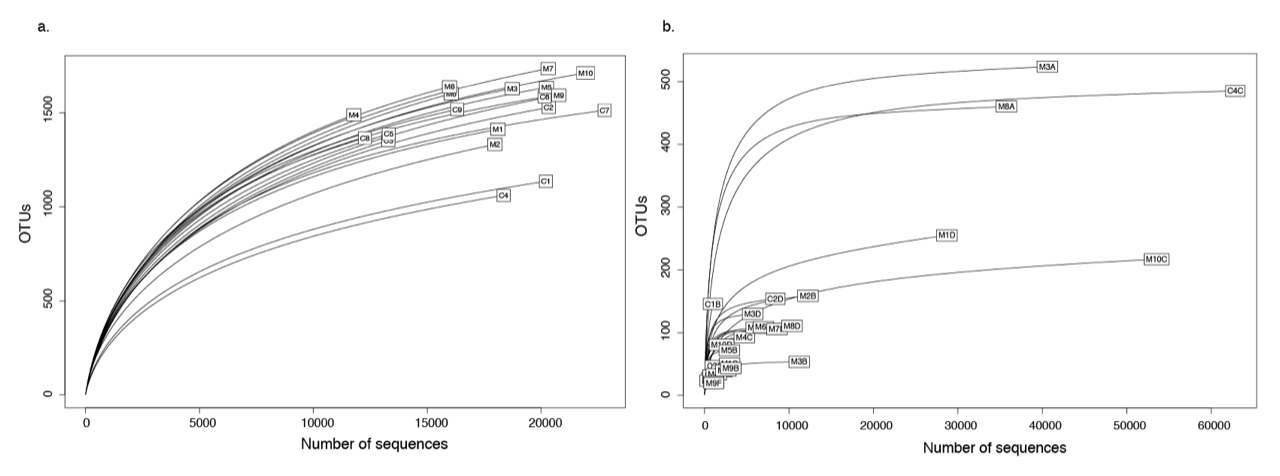
Figure S2. Rarefaction curves of sediment (≥10 cutoff dataset, a.) and foraminifera (≥5 cutoff dataset, b.) samples.

Table S1. Number of specimens per site / depth / species.

| **Site** | **Species** | **Number of specimens per depth (cm)** | | | | | | | | | | **Total** |
| --- | --- | --- | --- | --- | --- | --- | --- | --- | --- | --- | --- | --- |
|  |  | 0-1 | 1-2 | 2-3 | 3-4 | 4-5 | 5-6 | 6-7 | 7-8 | 8-9 | 9-10 |  |
| Mokbaai | *Ammonia* sp. | 2 | 3 | 2 |  | 2 | 1 | 1 | 1 | 1 |  | 13 |
|  | *Elphidium* sp. | 1 |  |  | 2 |  | 1 | 1 | 1 | 1 | 2 | 9 |
|  | *Haynesina* sp. |  |  | 1 |  |  |  |  |  |  |  | 1 |
| de Cocksdorp | *Ammonia* sp. |  |  |  |  |  |  |  |  |  |  | 0 |
|  | *Elphidium* sp. | 2 | 1 | 1 | 1 |  |  |  |  |  |  | 5 |
|  | *Haynesina* sp. |  |  |  |  |  |  |  |  |  |  | 0 |

Table S2. Primers used in this study. Sequences from 5’ to 3’.

| **Primer name** | **Target region** | **Sequence** | **Reference** |
| --- | --- | --- | --- |
| 27F | 16S V1-V3 | AGAGTTTGATCMTGGCTCAG | Lane et al. 1991^1^ |
| 519R | 16S V1-V3 | GTATTACCGCGGCTGCTG | Leser et al. 2002^2^ |
| AprA-1-FW | *aprA* | TGGCAGATCATGATYMAYGG | Meyer & Kuever 2007^3^ |
| AprA-5-RV | *aprA* | GCGCCAACYGGRCCRTA | Meyer & Kuever 2007^3^ |
| cd3aF | *nirS* | GTS AAC GTS AAG GAY ACS GG | Michotey et al. 2000^4^ |
| R3cd | *nirS* | GAS TTC GGY TGS GTC TTG A | Throback et al. 2004^5^ |
| F1aCu | *nirK* (bacteria) | ATC ATG GTS CTG CCG CG | Hallin & Lindgren 1999^6^ |
| R3Cu | *nirK* (bacteria) | GCC TCG ATC AGY TTG TGG TT | Hallin & Lindgren 1999 ^6^ |
| AnirKa-58F | *nirK* (archaea) | ACB YTA TTC GGA AGY ACA TAC ACA | Lund et al. 2012^7^ |
| AnirKa- 579R | *nirK* (archaea) | GYM ATT CCG TAC ATK CCG GA | Lund et al. 2012^7^ |
| cnorB2f | *cnorB* | GAC AAG NNN TAC TGG TGG T | Braker & Tiedge 2003^8^ |
| cnorB6r | *cnorB* | GAA NCC CCA NAC NCC NGC | Braker & Tiedge 2003^8^ |
| qnorB2f | *qnorB* | GGN CAY CAR GGN TAY GA | Braker & Tiedge 2003^8^ |
| qnorB5r | *qnorB* | ACC CAN AGR TGN ACN ACC CAC CA | Braker & Tiedge 2003^8^ |
| DSRp2060F | *dsrB* | CAA CAT CGT YCA YAC CCA GGG | Geets et al. 2006^9^ |
| DSR4R | *dsrB* | GTG TAG CAG TTA CCG CA | Wagner et al. 1998^10^ |
| amoA-1F | *amoA* (*β*-proteobacteria) | GGG GTT TCT ACT GGT GGT | Rotthauwe et al. 1997^11^ |
| amoA-2R | *amoA* (*β*-proteobacteria) | CCC CTC KGS AAA GCC TTC TTC | Rotthauwe et al. 1997^11^ |
| amoA-3F | *amoA* (*γ*-proteobacteria) | GGT GAG TGG GYT AAC MG | Purkhold et al. 2000 |
| amoB-4R | *amoA* (*γ*-proteobacteria) | GCT AGC CAC TTT CTG G | Purkhold et al. 2000 |
| CrenamoA-23F | *amoA* (Crenarchaeota) | ATG GTC TGG CTW AGA CG | Tourna et al. 2008 |
| CrenamoA-616R | *amoA* (Crenarchaeota) | GCC ATC CAT CTG TAT GTC CA | Tourna et al. 2008 |

Table S3. 20 most abundant OTUs in foraminifera and sediment samples including relative abundance (average % across all samples ± standard deviation)

|  | **Foraminifera** | **RA %** ± **SD** | **Sediment** | **RA %** ± **SD** |
| --- | --- | --- | --- | --- |
| 1 | Campylobacteria; *Sulfurovum* | 9.2 ± 14.6 | Campylobacteria; *Sulfurovum* | 14.8 ± 8.0 |
| 2 | Deltaproteobacteria; *Desulfobacula* | 5.4 ± 11.4 | Bacilli; *Paenisporosarcina* | 2 ± 3.7 |
| 3 | Chloroplast | 5.5 ± 9.6 | Deltaproteobacteria; Sva1033 | 1.9 ± 1.5 |
| 4 | Campylobacteria; *Arcobacter* | 3.4 ± 6.9 | Gammaproteobacteria; *Candidatus_Thiobios* | 1.8 ± 0.7 |
| 5 | Alphaproteobacteria; *Candidatus_Megaira* | 3.2 ± 17 | Gammaproteobacteria; B2M28 | 1.8 ± 1.1 |
| 6 | Chloroplast | 3.1 ± 8.8 | Chloroplast | 1.6 ± 1.3 |
| 7 | Campylobacteria; *Arcobacter* | 2.5 ± 3.5 | Actinobacteria; Actinomarinales | 1.3 ± 0.7 |
| 8 | Campylobacteria; *Arcobacter* | 1.8 ± 2.7 | Campylobacteria; *Sulfurovum* | 1.2 ± 0.9 |
| 9 | Gammaproteobacteria; *Candidatus_Thiobios* | 1.6 ± 5.3 | Deltaproteobacteria; Sva1033 | 1.1 ± 0.7 |
| 10 | Chloroplast | 1.5 ± 4.2 | Gammaproteobacteria; *Candidatus_Thiobios* | 1 ± 0.4 |
| 11 | Deltaproteobacteria; *Desulfosarcina* | 1.4 ± 2.6 | Gammaproteobacteria; *Sedimenticola* | 1 ± 0.5 |
| 12 | Deltaproteobacteria; *Desulfobulbus* | 1.3 ± 6.6 | Gammaproteobacteria; uncultured genus | 1 ± 0.4 |
| 13 | Bacteroidia; Crocinitomicaceae | 0.5 ± 1.8 | Gammaproteobacteria; *Thiogranum* | 0.9 ± 0.5 |
| 14 | Bacteroidia; *Candidatus_Amoebophilus* | 0.4 ± 2 | Alphaproteobacteria; *Loktanella* | 0.8 ± 0.5 |
| 15 | Deltaproteobacteria; Desulfobacteraceae | 0.4 ± 0.7 | Bacteroidia; Cyclobacteriaceae | 0.8 ± 0.6 |
| 16 | Deltaproteobacteria; Desulfobacterales | 0.4 ± 0.9 | Bacilli; *Planococcus* | 0.7 ± 2 |
| 17 | Deltaproteobacteria; *Desulforhopalus* | 0.3 ± 0.9 | Alphaproteobacteria; *Methyloceanibacter* | 0.7 ± 0.3 |
| 18 | Deltaproteobacteria; *Desulfopila* | 0.2 ± 0.8 | Gammaproteobacteria; Woeseia | 0.7 ± 0.5 |
| 19 | Bacteroidia; *Lutimonas* | 0.3 ± 0.8 | Dehalococcoidea; MSBL5 | 0.7 ± 1.1 |
| 20 | Deltaproteobacteria; *Desulfopila* | 0.1 ± 0.6 | Chloroplast | 0.6 ± 0.7 |

RA: relative abundance, SD: standard deviation

Table S4 10 most abundant Chloroplast OTUs in foraminifera (comprehending 69.1-89.1% of all reads in this class) and their closest NCBI BLAST match (excluding uncultured/environmental sample sequences) including identity score. Relative abundance given as an average % (± standard deviation) per species and site. Date of BLAST search: 10.04.2019.

| **Foraminifera** | | | | | | | |
| --- | --- | --- | --- | --- | --- | --- | --- |
|  |  |  | ***Elphidium* sp.** | | ***Ammonia* sp.** | ***Haynesina* sp.** |  |
| **OTU** | **Closest NCBI BLAST match** | **Blast ID %** | **C**  **%** | **M**  **%** | **M**  **%** | **M**  **%** |  |
| 1 | Virgulinella fragilis chloroplast (JN207201) | 99.6 | 73.6 ±42 | 44.4 ± 39 | 33.3 ± 39 | 87.9 |  |
| 2 | Triceratium dubium chloroplast (NC_038002) | 98.2 | 1.8 ± 3 | 15.3 ± 33 | 14.6 ± 29 | 0 |  |
| 3 | Bacterium WHC1-2 (JQ269270) | 97.7 | 1.5 ± 2 | 4.1 ± 6 | 8.8 ± 24 | 0.6 |  |
| 4 | Extubocellulus spinifer chloroplast (FJ002230) | 97.7 | 0.1 ± 0.2 | 11.1 ± 33 | 3.9 ± 8 | 0 |  |
| 5 | Ditylum brightwellii chloroplast (FJ159132) | 98.6 | 0.1 ± 0.2 | 3 ± 6 | 0 | 0 |  |
| 6 | Fragilariopsis cylindrus chloroplast (FJ002238) | 97.7 | 1.9 ± 4 | 0 | 5.1 ± 17 | 0 |  |
| 7 | Bacillaria paxillifer chloroplast (AJ536452) | 97.0 | 0.2 ± 0.3 | 0.2 ± 0.5 | 0.6 ± 2 | 0 |  |
| 8 | Nitzschia palea chloroplast (MH113811) | 98.4 | 0 | 11.1 ± 33 | 2 ± 7 | 0 |  |
| 9 | Plagiogramma staurophorum chloroplast  (NC_037995) | 96.9 | 0.4 ± 0.6 | 0 | 0.4 ± 1 | 0 |  |
| 10 | Delphineis sp. CCMP1095, (FJ002236) | 92 | 0 | 0 | 0.3 ± 1 | 0 |  |
| **Total %** | | | **79.5** | **89.1** | **69.1** | **88.5** |  |

C: de Cocksdorp, M: Mokbaai

Table S5 10 most abundant Chloroplast OTUs in sediment samples (comprehending 85.4-93.4% of all reads in this class) and their closest NCBI BLAST match (excluding uncultured/environmental sample sequences) including identity score. Relative abundance given as an average (± standard deviation) for all samples per site.

| **Sediment** | | | | |
| --- | --- | --- | --- | --- |
| **OTU** | **Closest NCBI BLAST match** | **Blast ID %** | **C %** | **M %** |
| 1 | Navicula phyllepta chloroplast (FJ002222) | 99.3 | 28.8 ± 16 | 32.9 ± 7 |
| 2 | Guinardia striata chloroplast (NC_037998) | 98.6 | 16.1 ± 14 | 11.6 ± 3 |
| 3 | Triceratium dubium chloroplast (NC_038002) | 98.6 | 18.1 ± 18 | 5.7 ± 3 |
| 4 | Plagiogramma staurophorum chloroplast (NC_037995) | 96.7 | 0.8 ± 2 | 15.1 ± 5 |
| 5 | Bacillaria paxillifer chloroplast (AJ536452) | 97.9 | 2.7 ± 2 | 12.9 ± 4 |
| 6 | Minidiscus trioculatus chloroplast (FJ002231) | 99.6 | 8.4 ± 5 | 7.2 ± 3 |
| 7 | Chaetoceros calcitrans chloroplast (FJ002215) | 99.8 | 7.3 ± 4 | 4.1 ± 3 |
| 8 | Eunotia naegelii chloroplast (KF733443) | 97.5 | 0.1 ± 0.2 | 1.9 ± 1 |
| 9 | Extubocellulus spinifer (FJ002230) | 99.5 | 2.5 ± 2 | 0.9 ± 1 |
| 10 | Biddulphia tridens chloroplast (NC_038007) | 97.9 | 0.6 ± 1 | 1.1 ± 1 |
| **Total %** | | | **85.4** | **93.4** |

C: de Cocksdorp, M: Mokbaai

Table S6. Average functional gene copy numbers of N and S cycle genes (± standard deviation) in sediment samples and inside the foraminiferal cells.

| **Gene**​ | **Function**​ | **Average gene copies/g sediment**​ | | **Average gene copies/foram cell**​ | | | |
| --- | --- | --- | --- | --- | --- | --- | --- |
|  |  | **C M** | | **C** | **M** | | |
|  |  |  |  | **E** | **E A H** | | |
| *amoA*  (β-Proteobacteria)​​ | NH_4_^+^to NH_2_OH ​ | 1.0 × 10^4^ ± 1.3 × 10^4^ | 1.8 × 10^4^ ±  1.2 × 10^4^ | NA | NA | NA | NA |
| *amoA*  ​  (γ-Proteobacteria)​ | NH_4_^+^to NH_2_OH ​ | 7.6 × 10^4^ ±  3.8 × 10^4^ | 1.4 × 10^5^ ±  5.8 × 10^4^ | NA | NA | NA | NA |
| Cren*amoA*​  (Crenarchaeota)​ | NH_4_^+^to NH_2_OH ​ | 1.4 × 10^1^ ± 1.1 × 10^1^ | 1.6 × 10^1^​ ±  9.3 | NA | NA | NA | NA |
| *nirS*​  (Bacteria)​ | NO_2_^-^ to NO | 2.8 × 10^5^ ±  1.7 × 10^5^ | 4.8 × 10^5^ ±  2.9 × 10^5^ | NA | NA | NA | NA |
| *nirK*​  (Bacteria)​ | NO_2_^-^ to NO | 7.8 × 10^5^ ± 4.5 × 10^5^ | 1.6 × 10^6^ ± ​  1.2 × 10^6^ | 3.4 × 10^2^ ± ​  3 × 10^2^ | 2.8 × 10^2^ ± ​  1.6 × 10^2^ | 3.5 × 10^2^ ± ​  1.6 × 10^2^ | 3.1 × 10^2^ |
| *AnirK*​  (Archaea)​ | NO_2_^-^ to NO | 1.2 × 10^-1^ ± 3.1 × 10^-1^ | 3.7 × 10^-2^ ±  3.5 × 10^-2^ | NA | NA | NA | NA |
| *cnorB*​  (Bacteria)​ | NO to N_2_O​ | 1.1 × 10^6^ ± 7.7 × 10^5^ | 1.6 × 10^6^​ ±  9.4 × 10^5^ | 9.5 × 10^2^ ± ​  1.9 × 10^3^ | 1.3 × 10^3^ ± ​  3.7 × 10^3^ | 3.7 × 10^3^ ± ​  3.9 × 10^3^ | 1.0 × 10^4^ |
| *qnorB*​  (Bacteria)​ | NO to N_2_O​ | 9.6 × 10^5^ ± 8.8 × 10^5^ | 1.6 × 10^6^ ±  1.4 × 10^6^ | NA | NA | NA | NA |
| *aprA*  (Bacteria) | APS to AMP + SO_3_^2−^ | 2.1 × 10^7^ ±  1.4 × 10^7^ | 2.9 × 10^7^ ±  1.8 × 10^7^ | 5.0 × 10^2^ ± ​  4.6 × 10^2^ | 1.0 × 10^3^ ± ​  1.5 × 10^3^ | 2.3 × 10^2^ ± ​  2.4 × 10^2^ | 8.4 × 10^1^ |
| *dsrB*  (Bacteria) | SO_3_^2−^ to H_2_S | 4.1 × 10^6^ ±  2.7 × 10^6^ | 7.8 × 10^6^ ±  6.0 × 10^6^ | 3.8 × 10^2^ ± ​  4.7 × 10^2^ | 1.9 × 10^3^ ± ​  2.9 × 10^3^ | 6.5 × 10^1^ ± ​  7.2 × 10^1^ | NA |

C: de Cocksdorp, M: Mokbaai, E: *Elphidium* sp., A: *Ammonia* sp., H: *Haynesina* sp., NA: Below the detection limit of the qPCR assay.

**References**

1. Lane, D.J. (1991). 16S/23S rRNA sequencing. In: Nucleic acid techniques in bacterial systematics. Stackebrandt, E., and Goodfellow, M., eds., John Wiley and Sons, New York, NY, pp. 115-175.
2. Leser, T., Amenuvor, J., Jensen, T., Lindecrona, R., Boye, M. and Moller, K. (2002). Culture-Independent Analysis of Gut Bacteria: the Pig Gastrointestinal Tract Microbiota Revisited. *Applied and Environmental Microbiology*, 68(2), pp.673-690
3. Meyer, B. and Kuever, J. (2007). Molecular analysis of the diversity of sulfate-reducing and sulfur-oxidizing prokaryotes in the environment, using *aprA* as functional marker gene. *Applied and Environmental Microbiology*, 73 (23) pp. 7664-7679.
4. Michotey, V., Mejean, V. & Bonin, P. (2000) Comparison of Methods for Quantification of Cytochrome cd1-Denitrifying Bacteria in Environmental Marine Samples. *Appl. Environ. Microbiol.* 66. pp. 1564–1571.
5. Throbäck, I. N., Enwall, K., Jarvis, A. & Hallin, S. (2004). Reassessing PCR primers targeting nirS, nirK and nosZ genes for community surveys of denitrifying bacteria with DGGE. *FEMS Microbiol. Ecol.* 49, pp. 401–417.
6. Hallin, S. & Lindgren, P. E. (1999). PCR detection of genes encoding nitrite reductase in denitrifying bacteria. *Appl. Environ. Microbiol.* 65, pp. 1652–1657.
7. Lund, M. B., Smith, J. M. & Francis, C. A. (2012). Diversity, abundance and expression of nitrite reductase (nirK)-like genes in marine thaumarchaea. *ISME J.* 6, pp. 1966–1977.
8. Braker, G. & Tiedje, J. M. (2003). Nitric oxide reductase (norB) genes from pure cultures and environmental samples. *Appl. Environ. Microbiol.* 69, pp. 3476–3483.
9. Geets, J. *et al*. DsrB gene-based DGGE for community and diversity surveys of sulfate-reducing bacteria. *J. Microbiol. Methods* 66, pp. 194–205.
10. Wagner, M., Roger, A. J., Flax, J. L., Brusseau, G. A. & Stahl, D. A. (1998). Phylogeny of dissimilatory sulfite reductases supports an early origin of sulfate respiration. *J. Bacteriol.* 180, pp. 2975–2982.
11. Rotthauwe, J., Witzel, K. & Liesack, W. (1997). The ammonia monooxygenase structural gene amoA as a functional marker: molecular fine-scale analysis of natural ammonia-oxidizing populations. *Appl. Envir. Microbiol.* 63, pp. 4704–4712.
12. Purkhold, U. *et al*. (2000)*.* Phylogeny of All Recognized Species of Ammonia Oxidizers Based on Comparative 16S rRNA and amoA Sequence Analysis: Implications for Molecular Diversity Surveys. *Appl. Environ. Microbiol.* 66, pp. 5368–5382.
13. Tourna, M., Freitag, T. E., Nicol, G. W. & Prosser, J. I. (2008). Growth, activity and temperature responses of ammonia-oxidizing archaea and bacteria in soil microcosms. *Environ. Microbiol.* 10, pp. 1357–1364.
